# “Don’t Look Up” Your Science—Herd Immunity or Herd Mentality?

**DOI:** 10.3390/microorganisms10071463

**Published:** 2022-07-20

**Authors:** Botond Z. Igyártó

**Affiliations:** Department of Microbiology and Immunology, Thomas Jefferson University, Philadelphia, PA 19107, USA; botond.igyarto@jefferson.edu

**Keywords:** COVID-19, pandemic, vaccines, herd immunity

## Abstract

This analysis piece will attempt to examine some of the critical pandemic-related measures implemented in the United States from an immunological perspective and pinpoint caveats that should have been considered before their implementation. I also discuss alternative measures grounded in scientific data that were not thoroughly explored and likely could have helped fight the pandemic.

## 1. How Much of a Threat Is/Was SARS-CoV-2?

The authorities in the US implemented strict lockdowns and restrictive blanket measures at the beginning of the pandemic in response to many unknown characteristics of the novel coronavirus strain, SARS-CoV-2 (severe acute respiratory syndrome coronavirus 2), such as spread, infectivity, target population, severity of disease caused by it, fatality rate, etc. These measures were meant to save lives directly and indirectly by reducing spread and protecting the healthcare system from being overwhelmed. However, these generalized restrictive measures remained in place even after new information became available, including which demographics had a high risk of becoming seriously sick and dying. In the following section, I will present data that would have supported the implementation of targeted restrictions, pinpoint some of the inaccuracies of COVID-19 (coronavirus disease 2019) statistics, and discuss them with a consideration of how influenza seasons are managed and quantified. 

Crucial parameters for precaution measures are the infection rate and especially the fatality rate. The infection fatality rate means the likelihood of death if you become infected. This is difficult to determine accurately, especially because, as we will see later, it is hard to define who died from COVID or with COVID, and depends on many factors. However, the consensus for healthy children and young adults is that this number, even for the more pathogenic earlier SARS-CoV-2 variants, is miniscule. The infection fatality rate is lowest among 5–14-year-old children and teenagers (~0.001%), then slowly increases with age through the 60–64 age group (~0.4%). With the 65–69 age group, the rate rises sharply (~1%) and could reach a few percent (~3%) around the age of 80. People aged 80 and over have more than an 8% chance of dying [1,2,3]. Thus, if you are elderly with other health issues or you are obese, etc., you are at higher risk of developing severe COVID with a fatal outcome. However, the same is valid for other respiratory infections, including respiratory syncytial virus and seasonal influenza [4,5,6]. Nevertheless, the authorities applied blanket measures. The data, however, support measures that would focus resources on protecting vulnerable people and leave everyone else to live a relatively undisturbed life. These generalized restrictive measures continued to be pushed even with the novel and less pathogenic variant of the SARS-CoV-2, Omicron [7,8], and doctors were pressured, against their own first-hand experience, not to call the new variant mild [8]. 

The authorities further substantiated strict blanket measures and lockdowns at the beginning of the pandemic with that if restrictions were not implemented, then SARS-CoV-2 would overwhelm the healthcare system. So, as we often hear from the experts, we must “flatten the curve.” However, the original two weeks to flatten the curve implemented at the beginning of the pandemic with the Wuhan variant turned into months/years, and the restrictions were not adjusted even after we learned much about this new coronavirus strain.

Despite the use of vaccines and available standards of care, major hospitals are regularly overwhelmed during seasonal influenza, but no drastic measures are taken to reduce the burden [9,10,11]. Unfortunately, it is impossible to directly compare the burden caused by the two viruses on the healthcare system because we do not have the reliable data to do so. We never mass-tested people for influenza, counting and including everyone who tested positive in the statistics, as was the case for SARS-CoV-2. The Centers for Disease Control and Prevention (CDC) only estimates how many adults become infected, hospitalized, or die yearly from/with influenza [12,13]. Some of the challenges that the CDC is admittedly facing in counting influenza-associated deaths are the following: “the sheer volume of deaths to be counted; the fact that not everyone that dies with an influenza-like illness is tested for influenza; and the fact that influenza-associated deaths are often a result of complications secondary to influenza and underlying medical problems, and this may be difficult to sort out [12].” Based on this reasoning, COVID-related death statistics cannot be accurate. Indeed, for COVID statistics, the criteria to be counted are superficial, and almost anyone who tested positive but died from other causes and underlying conditions is included [14,15]. Furthermore, out of roughly 50% of people who died with/from COVID, influenza/pneumonia was listed as a comorbidity [16]. While the US officially did not have an influenza season, using the logic applied for generating COVID statistics, these data could also be interpreted as these people dying because of influenza/pneumonia. Similar strategies are used to track COVID hospitalization numbers. Everyone who tested positive for SARS-CoV-2 at hospital check-in, but was admitted for other health reasons, counted towards the numbers of COVID hospitalizations [17,18]. Whether the unreliable COVID tests [19,20,21] and COVID-related incentives provided to hospitals by the federal government further contributed to the erratic statistics, remains to be determined [22]. Thus, the infection fatality rate data support protective measures geared towards people at risk, and COVID-related statistics should be revised. Further studies will be needed to determine whether the COVID statistics and heavy media display of graphic material contributed to increased fear and anxiety in the population and better adherence to restrictions and vaccination requirements [23].

## 2. How Not to Vaccinate Ourselves out of a Pandemic 

Scientists have been working on an effective universal influenza (RNA virus) vaccine for decades. We still do not have one, and we have not developed herd immunity from natural infections. As possible reasons for failure, we could mention that: 1. RNA viruses frequently mutate to generate new variants to evade pre-existing immunity; 2. It is hard to make the immune system to react to conserved viral elements; and 3. For ill-defined reasons many people do not develop long-lasting immune memory after infection or vaccination [24,25,26]. SARS-CoV-2 is also an RNA virus, and therefore prone to mutations, though, it was initially thought to mutate at a slightly lower rate than other RNA viruses [27]. Thus, through mutations, it is expected to evade pre-existing immunity sooner or later, especially if we focus the vaccine on a single antigenic target such as the spike protein that is critical for the virus to propagate. The so-called common human coronaviruses that contribute to 15–30% of cases of common colds in humans, also belong to this virus family, and can sometimes cause life-threatening lower respiratory tract infections in infants, elderly people, or immunocompromised patients [28]. However, mainly for the above-mentioned reasons, no serious attempts have been made by scientists to generate a vaccine targeting these virus strains. Therefore, from a scientific perspective, thinking that we can make an effective vaccine against SARS-CoV-2 by focusing on a constantly changing protein in a year or so to end the pandemic lacked a solid scientific rationale. Nevertheless, if we still wanted to fight this pandemic with a vaccine, then the “old school” vaccine platform, relying on whole killed/live attenuated virus, that helped us to control or eradicate other viruses, such as smallpox, polio, yellow fever, rabies, etc., should have been given priority [29]. The approach of a killed or live-attenuated SARS-CoV-2 vaccine formulation delivered intranasally would have been the most supported approach by a strong scientific rationale. From an immunological perspective, this vaccine formulation would have contained all the viral proteins and molecular determinants that the immune system could react to, providing the most comprehensive possible protection, even from subsequent variants [30]. Furthermore, a vaccine delivered intranasally, unlike the present ones injected intramuscularly, would have likely generated protective immunity at the mucosal sites (airways) where we are exposed to the virus, and where we need it the most [31,32,33,34]. While the generation and optimization of an intranasal vaccine could take longer than the mRNA-LNP (mRNA combined with lipid nanoparticles) vaccine and possible adverse reactions from disease-causing potential apply for a live-attenuated vaccine [35], the fact that these vaccines could have been made almost anywhere, would have increased the likelihood of fast, even, and fair worldwide distribution at a more affordable price. 

The caveats discussed above must have been apparent to the companies designing these vaccines. Moving forward for example with the untested mRNA-LNP platform focusing on one viral protein was therefore likely rationalized by the comparatively fast rate at which they could be developed. The idea is that a suboptimal but profitable vaccine in terms of immunological effectiveness is better than an optimal vaccine that will not beat its competition to market. Unfortunately, as we will see later, the drawbacks of the vaccines were neither accurately presented to the public, nor adequately scrutinized by the scientific community. Their unjustified touting by “expert” voices resulted in blind group adherence to a flawed strategy for combating the pandemic. In the future, we need to move away from a system incentivizing profit over the best healthcare product and demand transparency in the communication of clinical data [36,37]. The Food and Drug Administration (FDA) and CDC or, even better, an independent entity should demand from the companies interested in developing a vaccine or healthcare product to present a plan in the form of a scientific proposal. Very much like scientists competing for grants, the companies’ proposals would be ranked based on scientific merit and feasibility, with the selected proposals supported and fast-tracked through programs such as Operation Warp Speed [38].

## 3. All That Glitters Is Not Gold

Selecting, supporting, and promoting the mRNA-LNP platform, which has never been used in humans before and for which we lack long-term safety data over well-established killed/attenuated vaccine platforms to fight COVID, lacked solid scientific rationale. In this section, we will take a closer look at the clinical trial data with the mRNA-LNP platform, briefly discuss how this platform works and present some of its drawbacks. 

The mRNA-LNP-based vaccine clinical trials compared the number of COVID cases in healthy individuals divided into vaccinated vs. control (placebo) groups. The placebo group received pure saline, and no control group injected with empty LNPs was used. A case was defined as an individual who experienced symptoms and had a positive test for SARS-CoV-2 infection [39]. The incidence of severe disease and death, as endpoints, were not considered [39,40,41]. These trials reported that the mRNA-LNP vaccines are around 95% effective in relative risk reduction (RRR) [42]. However, the companies failed to report the absolute risk reduction (ARR) and did not make the complete clinical trial data publicly available [36,37,43]. While the RRRs give a percentage reduction in one group (vaccinated) compared to another (placebo), the ARRs show the actual difference in risk between one group and another. The RRR for the mRNA-LNP vaccines was ~95%, while the ARR was later estimated to be ~1%. The authorities would probably have had a hard time convincing people to take the vaccine if they had stated that the shot reduces your risk of becoming infected by only ~1% or that you need to give ~100 people shots to prevent one infection. “Omitting ARR findings in public health and clinical reports of vaccine efficacy is an example of outcome reporting bias, which ignores unfavorable outcomes and misleads the public’s impression and scientific understanding of a treatment’s efficacy and benefits. Furthermore, the ethical and legal obligation of informed consent requires that patients are educated about the risks and benefits of a healthcare procedure or intervention [44]”.

The novelty of the mRNA-LNP platform is that the body makes proteins using the mRNA as a template. Thus, vaccine companies do not have to produce the proteins upfront. The component of this platform that supports potent immune responses is the lipid nanoparticles’ (LNP) ionizable lipid (a synthetic molecule with a long life span; [45]), which is shown to be highly inflammatory [46,47,48] and the likely driver of many of the documented side effects of this platform. If you take these LNPs and mix them with proteins, you obtain similar immune responses to the mRNA-LNP [49]. Thus, the critical component here is the LNP with ill-defined biological effects but strong adjuvant and inflammatory properties, which likely also contribute to the immune system’s reprogramming [50,51]. While the positive aspect of this platform is widely publicized, the authorities seem to not be concerned by the severe side effects and thousands of deaths reported so far for this platform in the Vaccine Adverse Event Reporting System (VAERS) database [52]. This might be because it is hard to establish direct causation in most cases, which becomes almost impossible with time. The VAERS is known to underreport certain cases [53]. Nevertheless, the number of already reported death cases surpasses the ones reported for all the other vaccines pooled together. This should raise a red flag at the regulatory agencies and trigger some sort of investigation, especially since more and more peer-reviewed and preprint case reports document the existence of the short- and long-term side effects of these vaccines [54]. These include, but are not limited to, fatal and non-fatal cases affecting the cardiovascular and nervous systems. Autoimmune cases targeting different organs, hepatitis, virus reactivation, multisystem inflammatory syndrome cases, etc., were also reported [54,55]. Whether the documented side effects are linked to the highly inflammatory properties of the LNPs, the autoimmune reactions targeting the spike protein-expressing cells [47], the pathogenicity of the spike protein coded by the vaccines, or the combination of these and other factors, remains to be determined. The spike protein coded in these vaccines is stabilized in a pre-fusion form and contains a membrane anchor sequence. These modifications might make it less pathogenic than the wild-type viral spike protein [56,57], but since the vaccine components can directly or indirectly reach almost any organ in the body [46,58,59,60,61], people exposed to multiple shots might be at higher risk of developing the presented side effects. A recent study revealed another potential, unexpected problem with the mRNA-LNP platform. This study showed that the vaccine mRNA could be reverse transcribed into DNA in a human hepatic cell line in vitro [62]. It remains to be determined whether this can be observed in vivo and at physiologically relevant levels [63]. However, if this phenomenon exists combined with genomic insertion, it might bear serious health concerns, especially if reproductive cells are affected.

The benefit of COVID vaccines probably outweighs the risk of serious side effects for anyone with major underlying health conditions, especially the elderly, but certainly not for healthy children, teenagers, and young adults. While based on present legislation, companies cannot be held responsible for the harm caused by their vaccines, allocating a small percentage of their profits to a fund that would be used to compensate people for their suffering and loss, or alternatively, selling the vaccine in critical times at production costs as AstraZeneca did [64] would show goodwill and might increase people’s trust in these products. 

## 4. SARS-CoV-2 Vaccines and Herd Immunity—A Fictional Romance 

After the partial clinical trial data became public, the mRNA-LNP vaccines were marketed by government officials and experts, who suggested that if you take the vaccine, you will not get infected; therefore, you will protect yourself and the people around you. This narrative, however, was formulated on the pretext that the vaccines provided “sterilizing” immunity from SARS-CoV-2. Nevertheless, experts suggested that we can reach herd immunity if ~60% of the world population become immunized. After the “breakthrough” infections started to become apparent and later proved that vaccines do not stop people from getting infected, or spreading the virus, and that vaccinated individuals can contain similar levels of viral loads as unvaccinated people [65], the narrative changed, stating that at least you are less likely to get seriously ill and die. Experts responded to these data by bumping up their estimates on the percent of the population that must be vaccinated to reach herd immunity. To achieve the very high numbers (80–90%), some experts suggested that teenagers and, later, children must be included in the pool and get vaccinated. The idea behind herd immunity is that if enough people are immune, then the pathogen will not find enough susceptible hosts through which to replicate and spread and will eventually die out. Thus, one might wonder, if the vaccines do not provide sterilizing immunity, and people can still become infected and spread the virus, is it even theoretically possible to reach herd immunity? Data from countries with high vaccination rates prove that these vaccines, even combined with endless boosters, will not achieve herd immunity nor protect us from new variants and waves. The uncontrolled spread of Omicron through the US/world further highlights the subpar performance of the vaccines in preventing viral infections and spread. More than 70% of the first Omicron cases reported by the CDC were fully vaccinated [66]. The boosters based on the spike protein from the original virus variant, as expected and indicated, are becoming less and less effective with the new variants but might still provide some benefits in preventing severe illness and death. These benefits are probably provided by vaccine-induced antibodies circulating in the bloodstream and preventing systemic viral spread. However, the same antibodies might also contribute to an increased risk of antibody-mediated enhancement upon exposure to the virus [67,68]. While some research supports the generation of memory responses [69,70], the overwhelming majority shows a waning immunity, leading manufacturers and regulatory agencies to recommend frequent booster shots [71]. The need for continuous boosters targeting earlier variants also argues that the vaccines do not induce long-lasting memory responses or that natural infections cannot reactivate them for unknown reasons.

## 5. Alternative Measures to Vaccines to Fight the Pandemic

Bias towards vaccines as the single most crucial tool to fight the pandemic was evident from the beginning. However, these vaccines do not prevent infections and provide little protection from long COVID [72], and even if we succeeded in injecting the entire world population, we would have likely put an even stronger evolutionary pressure on the virus to mutate and evade the immune responses triggered by the vaccine. The best example of how non-sterilizing vaccines can lead to more pathogenic variants is the vaccine generated to fight Marek disease in poultry [73,74]. This caveat and those discussed above highlight the lack of a solid scientific rationale to focus on vaccines targeting a single viral component. We have been fighting influenza for decades. Influenza induces short- and long-term symptoms and kills people in a similar way to COVID, and the same target population is at risk [75,76,77,78,79,80,81]. We have a standard of care in place with influenza symptoms and other respiratory diseases, some of which could have been better used to treat COVID. 

Efforts to repurpose drugs with high safety profiles could have been better supported and managed. Drug repurposing and developing novel therapeutics to treat COVID have been reviewed elsewhere [82]. Here, I will briefly discuss the efforts to repurpose two drugs, chloroquine (alone, or its hydroxy derivate, or combined with azithromycin) and remdesivir, to highlight possible concerns on how drugs become classified as effective by regulatory agencies. Chloroquine, hydroxychloroquine, and azithromycin are cheap and safe drugs taken by millions around the world daily for different inflammatory diseases and malaria. After some promising data on the use of these drugs to treat COVID [83], follow-up clinical trials failed to show their benefits in fighting COVID [84,85,86]; therefore, they were not implemented as the standard of care in Western countries. Chloroquine/hydroxychloroquine/azithromycin/ivermectin are still the primary go-to drugs in low-mortality-rate African countries to prevent and treat COVID [87,88,89,90]. Whether the low mortality rate in these countries is due to the use of these drugs or other factors, such as vaccination history [91,92,93], age, etc., remains to be determined [90]. In contrast, the previously untested remdesivir was fast-tracked and adopted as the standard of care despite opposing clinical trial outcomes [94]. One well-designed but maybe underpowered clinical trial did not reveal any statistically significant clinical benefits of remdesivir for treating severe COVID [95,96], and neither did the WHO Solidarity Trial [97]. In contrast, The National Institute of Allergy and Infectious Diseases (NIAID)-sponsored, somewhat controversial trial [98] found that remdesivir slightly reduced the duration of the disease (from 15 days to 11 days), though not mortality from COVID [99,100]. However, the already questionable benefits of using remdesivir might be even further diminished by reports of kidney and liver toxicity [101]. 

The monoclonal antibody therapy targeting the SARS-CoV-2 spike protein from Regeneron and others became available early in the pandemic. It was shown to be highly effective in preventing the development of severe illnesses and hospitalization without any significant side effects. This treatment option, which can now be easily administered subcutaneously [102], could likely have been made available to anyone who needed it for a fraction of the total vaccine costs. 

Early research showed that patients with severe COVID had low levels of vitamin D [103,104,105]. People with dark skin need more sunlight than people of Northern European ancestry to generate similar amounts of vitamin D, and they are disproportionally affected by COVID [106,107,108]. Therefore, simple lifestyle adjustments that involve nutrition, exercise, and supplements should probably have been implemented as a preventative measure against COVID. These measures would likely also constitute a long-term general solution for achieving a much healthier population that is more resistant to infections and chronic diseases.

## 6. Discredited Science

Science, in general, is still about questioning everything using common sense and data, but unfortunately not when it comes to pandemic-related measures. Knowingly risking their careers, funding, discreditation, etc., an increasing number of scientists decided to speak up on the COVID-related measures, that they felt lacked solid foundation [40,43,109]. However, their data, ideas, concerns were largely ignored or suppressed by officials [110] and labeled as fake science by the media. We have done a disservice to science by saying we “follow the science,” when what we mean is that we follow the handpicked, politicized, and popular science. Science is not about censoring everyone who does not agree with the official narrative. Scientific rigor requires considering opposing views even if they are not published in high-impact journals, or they do not come from “top/celebrity” scientists. Lately, even articles published in prestigious scientific journals have been censored by individuals working for social media platforms (Rapid Response: Open letter from The BMJ to Mark Zuckerberg) [111].

The best measures that we can take to limit disinformation and gain public trust in pharmaceutical products is not through censored science but through transparency and integrity. Here are a few examples that do not serve that purpose and further muddy the waters and fuel conspiracy theories: 1. Sending Dr. Peter Daszak, who subcontracted NIH money to the Wuhan Institute of Virology for coronavirus research, to investigate the possible lab leak theory [112]; 2. The fact that the FDA is estimating that it will take 55 to 75 years until all the documentation pertaining to the approval of the Pfizer COVID vaccine will be made public (in contrast it took the FDA roughly 100 days to approve the vaccine) [113,114]; 3. The FDA-approved Pfizer COVID vaccine is not widely available for research; and 4. For a virus that is everywhere, it is time for the CDC to stop restricting SARS-CoV-2 research to the limited numbers of biosafety level 3 facilities that exist in the US [115].

## 7. Layman’s Summary

The virus fatality rate did not support lockdowns, blanket restrictive measures, and non-selective mass vaccinations. There was no solid scientific rationale to adopt the untested mRNA-LNP platform over other well-established vaccine formulations to fight COVID. Scientists, experts, government officials, the media, and scientific journals, all contributed to suppressing alternative ideas on how to manage the pandemic. Some of these groups are still promoting the scientifically debunked idea that the present vaccines protect you from catching COVID and becoming sick [116], and that this is a pandemic of the unvaccinated [117]. Unfortunately, the virus is likely here to stay in one form or another, and we must learn to live with it. How effective the lockdowns and other restrictive measures were in reducing COVID deaths are still a matter of debate [118,119,120,121], but we likely lost, or we will lose a lot more people, from the direct and indirect effects of these measures. The excess of global deaths is often exclusively attributed to COVID [122]. However, these reports fail to factor in the direct and indirect effects of COVID-related restrictions. Many people died because they did not have access to healthcare during this period (they were afraid of going to the hospitals, or the hospitals did not accept them). Furthermore, a significant increase in suicide rates, drug and alcohol abuse, domestic violence, obesity, economic hardships, vaccination-related events, etc., likely contributed to excess deaths. If the vaccines do not prevent infections and spread, it is time to stop coercing people to get vaccinated, vilifying unvaccinated people, and giving extra perks to the vaccinated. It is neither feasible nor economically sustainable to “vaccinate” everyone every few months or perform continuous testing, and more importantly, there is no scientific rationale for doing so. We must focus our resources on the vulnerable population and provide them with efficient treatment options and possible preventative steps in the form of lifestyle changes, supplements, and effective, long-lasting, safe, and affordable vaccines.

## Statement

This is an opinion paper. The opinions expressed in the manuscript are mine and do not necessarily reflect the official position of Thomas Jefferson University nor the opinion of the MDPI group. It is imperative to emphasize that vaccines such as smallpox, polio, yellow fever, rabies, etc. have done amazing things for public health. Thus, this opinion article is only questioning the particularities of the current response to the COVID-19 pandemic, including the generation and use of novel vaccines and therapeutics that did not undergo careful long-term evaluation for both safety and efficacy.
